# Exercise for survivorship: outcomes associated with a community-based group exercise program for adolescent and young adult cancer survivors

**DOI:** 10.1007/s00520-026-10838-0

**Published:** 2026-06-06

**Authors:** Claire Munsie, Jo Collins, Jay Ebert, Megan Plaster

**Affiliations:** 1 Western Australian Youth Cancer Service, North Metropolitan Health Service, Perth, Australia; 2 School of Allied Health, Curtin University, Perth, Australia; 3 School of Human Sciences (Exercise and Sport Science), University of Western Australia, Perth, Australia; 4 Nursing Services, Perth Children’s Hospital, Perth, Australia; 5 Department of Haematology, North Metropolitan Health Service, Perth, Australia

**Keywords:** Adolescent and Young Adult, Survivorship, Community-based, Exercise

## Abstract

**Background:**

Adolescent and young adult (AYA) cancer survivors experience persistent physical and psychosocial effects after treatment. Despite evidence supporting exercise in adult cohorts, research specific to AYAs remains limited. This study examined physical and psychosocial outcomes associated with participation in a community-based, group exercise program.

**Methods:**

A pragmatic, single-arm, pre-post study design was used. AYAs aged 15–27 years within 2 years post-treatment completed a 12-week group exercise program in a community setting. Biweekly, individualized sessions incorporated aerobic, resistance, and flexibility training under an accredited exercise physiologist’s supervision. The primary outcome was predicted VO₂peak, with secondary outcomes including one-repetition maximum (1RM) strength, physical function, body composition, and quality of life (EORTC QLQ-C30, SF-36). Pre–post changes were analyzed using mixed-effects models adjusted for diagnosis.

**Results:**

Of 127 participants (mean age 21.4 ± 3.0 years; 51% male), 93 (73%) completed the program and final assessments. A significant improvement was observed in predicted VO₂peak (3.1 mL·kg⁻^1^·min⁻^1^, *p* < 0.001), all 1RM strength measures (leg press* p* < 0.001; chest press *p* < 0.001; seated row *p* < 0.001), physical function (push ups *p* < 0.001; sit ups *p* < 0.001; 5-rep sit-to-stand *p* < 0.001; grip strength *p *= 0.028), lean mass (*p* < 0.001), and quality of life across physical, role, and social domains (*p* < 0.001). Fat mass percentage (*p* = 0.002) and fatigue (*p* < 0.001) decreased. No adverse events occurred.

**Conclusion:**

Community-based, group exercise was safe and was associated with improvements in fitness, strength, and quality of life among AYA cancer survivors. Findings are consistent with a beneficial association between scalable, real-world exercise models and long-term survivorship outcomes. This trial was retrospectively registered (ACTRN12620000664943) on 10th June 2020.

**Supplementary Information:**

The online version contains supplementary material available at 10.1007/s00520-026-10838-0.

## Introduction

Between 2014 and 2018, 5302 adolescents and young adults (AYAs) aged 15–25 years were diagnosed with cancer in Australia [1]. Advances in treatment over the past two decades have improved 5-year survival rates in this population from 79 to 90% [1], creating a growing cohort of survivors now living with the persistent physical, psychological, and social sequelae of cancer and its treatment [2]. As this population enters adulthood, they face the potential risks of chronic disease, persistent fatigue, cardiovascular complications, and elevated psychological morbidity compared with their healthy peers [3, 4], placing considerable burden on both individuals and the healthcare system [5].

Exercise is recognized to be efficacious in counteracting cancer treatment-related toxicities, such as fatigue, peripheral neuropathy, and physical deconditioning, and in improving quality of life (QoL) in a range of cancer cohorts [6–8]. However, evidence in AYAs is lacking, with a dearth of large-scale, high-quality research evident [9, 10]. Current survivorship guidelines recommend a minimum of 150 min of combined, moderate-intensity aerobic with two resistance training sessions per week [11, 12]. Yet, only around half of AYA survivors meet these guidelines compared with 70% pre-diagnosis [13]. Additionally, a large cross-sectional survey reported that although 78% of AYAs expressed interest in cancer-specific exercise programs, only 5% had accessed such services during and following their cancer care [14]. Barriers to participation are multifactorial and include treatment-related side effects such as pain, fatigue, and nausea, as well as psychological, socioeconomic, and lifestyle disruptions [15]. These discrepancies between preferences, availability, and participation highlight the need for targeted, evidence-based programs tailored to the unique needs of this cohort.

The limited published studies in AYA survivors have demonstrated safety, as well as physical and psychological benefits associated with exercise participation [10]. However, they have been undertaken in small sample sizes, employ conflicting age definitions ranging from 15–25 to 15–39 years, have an underrepresentation of younger AYAs (15–25 years), include childhood cancer survivors, and lack scalable, standardized models [10]. While these programs are primarily individualized and are undertaken in clinical or home-based environments, community group-based programs may facilitate the transition from structured, supervised clinical care to independent exercise participation, which is often reported as a challenging process in this cohort. This study sought to evaluate outcomes associated with participation in a community-based, group exercise program for AYA cancer survivors, designed to support real-world survivorship integration. It was hypothesized that participation in this program would result in significant improvements in (1) cardiorespiratory fitness (predicted VO₂peak) and (2) an array of other strength, physical function and quality of life outcomes.

## Methods

### Study design

This study was developed and conducted within a community-based setting to complement existing standard of care practices. Accordingly, an effectiveness (pragmatic) design approach was employed [16]. This design accounts for external influences including patient, provider, and/or system-level factors that may moderate the intervention effects. Incorporating these contextual variables ensures that the evaluation reflects the real-world conditions under which the intervention was implemented and integrated into standard care [16].

Several provider- and system-level factors were intentionally embedded to reflect real-world delivery conditions. At the provider level, the same AEP responsible for recruitment also supervised the program, mirroring the clinical continuity typical of survivorship services. Group sizes, session duration, and exercise prescription were individualized rather than standardized, and informal pre-program group activities were incorporated to foster cohesion [17], reflecting the flexibility of community clinical practice. At the system level, participants were identified from the Western Australian Youth Cancer Service (WAYCS) end-of-treatment pathway and required medical clearance from their treating clinician prior to enrolment, integrating the program within existing clinical decision-making. The community gymnasium setting and use of existing oncology referral networks were selected to reflect conditions under which a scaled service would operate. Provider-level factors, such as AEP experience and session fidelity, and system-level factors, such as referral rates and organizational capacity, were not systematically captured and may have moderated outcomes; this represents a limitation of the current design.

### Program development

Stakeholder consultation revealed that the existing Cancer Council Western Australia *Life Now* Exercise Program had primarily been utilized by older adults with comparatively low engagement among AYA cancer survivors and was not fit for purpose for this age group [18]. While *Life Now* is designed to address treatment-related side effects and enhance physical and psychosocial well-being in a community-based setting, adaptations were required to ensure developmental relevance. Consultation involved formal meetings and informal discussion with consumer advocates, AYA cancer clinicians (nurses, AEPs, AYA physicians), and Cancer Council WA Life Now program staff. Key barriers identified included the predominance of older adult participants; exercise content not calibrated to younger survivors’ fitness levels and developmental needs; and limited opportunity for peer connection with others at a similar life stage. Consequently, age-appropriate modifications were made in collaboration with key stakeholders resulting in the AYA *Life Now* Exercise Program, which incorporates tailored exercise prescription and targeted support to improve relevance and uptake.

### Participants and recruitment

Ethics approval was granted by Sir Charles Gairdner Hospital HREC (RGS0000001819). Potential participants were identified from the Western Australian Youth Cancer Service (WAYCS) “End of Treatment” database and through promotional materials distributed to oncology and allied health teams at local hospitals. To be included in this study, participants needed to be English-speaking AYAs aged 15–25 years at time of diagnosis and subsequent enrolment in this study, having completed their primary cancer treatment within the past 2 years. Participants were excluded from study inclusion if they presented with medical instability and deemed inappropriate to participate in moderate-intensity exercise by their treating clinician, had severe anemia or neutropenia, bone metastases, or lacked medical clearance. Participants deemed ineligible were offered ongoing one-to-one supervised exercise within the hospital environment as per current standard of care. Participants (initially via their legal guardians if < 18 years) were contacted by the WAYCS Accredited Exercise Physiologist (AEP) via phone and email and given 2 weeks to respond. Non-responders were followed up to clarify participation. Written informed consent was obtained following discussion with the participant and their guardian if appropriate prior to baseline assessments.

### Exercise intervention

Following baseline assessment (outlined below), participants completed a 12-week, bi-weekly, supervised group-based exercise program delivered in a community setting. Informal pre-program group activities were incorporated to foster social connection and facilitate group cohesion [17]. The community setting, a small, private gymnasium, was selected to minimize potential psychological distress associated with returning to a hospital environment and to better reflect real-world exercise participation. The exercise intervention was designed in accordance with national and international guidelines for exercise prescription in cancer survivors [11, 12]. Bi-weekly sessions were 60–90 min in duration, included up to ten AYAs per group, and were supervised by an AEP and three Master of Clinical Exercise Physiology students. Each session incorporated aerobic (steady-state and interval training), resistance, and flexibility exercises individualized to each participant’s needs based on their baseline assessment outcomes. Aerobic training was prescribed at 50–80% of predicted maximal heart rate or Borg rating of perceived exertion (RPE) 12–15, progressing to include intervals when participants achieved 20 min of moderate-intensity steady-state activity. Resistance training targeted seven to ten multi-joint exercises at 60–85% of one-repetition maximum (1RM), progressing from 2 × 10–12 repetitions to 4 × 8 repetitions by week 12. Flexibility training included 5 min of static stretching for major muscle groups.

### Outcome measures

Recruited participants underwent baseline assessment, as well as post-intervention evaluation (within 3 days of their final session), utilizing a series of standardized outcome measures. All assessments were conducted at The University of Western Australia, School of Human Science (Exercise and Sports Science) gymnasium. Safety screening was performed before each assessment, with modifications or exclusions applied where necessary. Full details of the pre-program medical clearance process, pre-assessment and pre-session safety screening procedures, and adverse event monitoring protocols are provided in Supplementary Material 1 [11, 19]. Outcome measures were selected to provide insight into the physical fitness, strength, body composition, and psychosocial well-being of this cohort.

### Assessment of cardiorespiratory fitness

Predicted VO₂peak was selected as the primary outcome given its established prognostic value in cancer survivorship. Post-diagnosis CRF is independently associated with all-cause mortality, cause-specific cardiovascular and cancer mortality, and long-term health burden in cancer survivors [20], and its use is consistent with prior exercise oncology trials in this population, enabling cross-study comparison [21]. A submaximal protocol was selected on clinical and safety grounds. Participants were recently post-treatment, presented with heterogeneous diagnoses and treatment exposures, and demonstrated variable baseline fitness levels; maximal exertion testing in this context carried elevated cardiovascular and musculoskeletal risk and was considered inappropriate without prior systematic cardiac screening beyond the scope of this study. It is acknowledged that CPET-based assessment requires specialized equipment and trained personnel, limiting scalability and reducing direct relevance to patients and service commissioners. Predicted VO₂peak was estimated from a submaximal cardiopulmonary exercise test (CPET) using heart rate extrapolation. Resting heart rate and blood pressure were recorded electronically prior to testing (Wahoo Tickr, Wahoo Fitness LLC, GA, USA). A target heart rate (THR) of *(220 − age)*[22] × *0.85* was used as the termination criterion unless the participant reached volitional exhaustion prior. In all cases, predicted VO₂peak was estimated using the submaximal extrapolation method [21]. Testing was performed on a front-access cycle ergometer (Exertech Ex-10, Repco Cycle Company, Huntingdale, Australia) using a ramped protocol starting at 20 W, with increments of 20 W per minute until THR or exhaustion. Heart rate and RPE [23] were recorded each minute, followed by a 3-min cool-down. Participants wore a non-rebreathing mouthpiece attached to a computerized gas analysis system (Universal Ventilation Meter, Vacumed, CA, USA; Ametek S-3A/1 and CD-3A analyzers, AEI Technologies, PA, USA) to measure minute ventilation (VE), respiratory exchange ratio (RER), and oxygen and carbon dioxide exchange at 15-s intervals. Predicted VO₂peak values were expressed in absolute (L/min) and relative (mL/kg/min) terms. Predicted VO₂peak was estimated using the slope of two submaximal VO₂-heart rate data points (steady-state HR 115–150 bpm), extrapolated to predicted maximal heart rate [24]. To replicate functional activities of daily living, participants also completed a 400-m timed-walk test [25]. This test was also used as a surrogate measure of CRF for participants deemed ineligible to complete CPET. Participants were required to walk ten laps of a marked out a 20-m distance at a pace that was sustainable for the entire test. Total time taken and RPE were recorded at the end of the test.

### Assessment of anthropometrics and body composition

Dual-energy X-ray absorptiometry (DXA) (Lunar Prodigy, GE Medical Systems) assessed lean muscle mass (LMM), fat mass (FM), body fat percentage (FM%), bone mineral content (BMC), and bone mineral density (BMD). Additionally, height, weight, body mass index (BMI), and waist/hip circumferences were collected using standard assessment techniques [26].

### Assessment of strength and functional capacity

Maximal upper and lower body strength were measured using standardized 1RM tests (leg press, chest press, seated row) [26]. Functional upper limb, trunk, and lower limb strength were assessed using a 30-s push up test, 30-s sit up test, and a timed five-repetition sit-to-stand (STS) test, respectively [26]. Both 30-s tests required participants to complete maximal repetitions within the 30-s timed period, while the five-repetition STS test recorded the fastest attempt of three trials. Finally, maximal hand grip strength of the dominant upper limb was also assessed (Jamar Plus Digital Dynamometer), with the best of three attempts included.

### Patient-reported outcome measures

Patient-reported outcome measures (PROMs) were employed to capture changes in psychosocial variables, fatigue, and patient-reported physical activity and QoL. Cancer-specific quality of life was captured using the European Organisation for Research and Treatment of Cancer Quality of Life questionnaire (EORTC QLQ-C30) [27]. Additional general health-related quality of life was captured using 36-item Short Form Health Survey to allow for potential comparison against aged-matched normative data [28]. Self-reported physical activity was captured using Godin Leisure-Time Physical Activity Questionnaire [29].

### Sample size calculation and statistical analysis

Sample size was calculated to detect a clinically meaningful improvement in VO₂peak consistent with findings from Atkinson et al. [30]. The estimated mean change was 2.85 mL·kg⁻^1^·min⁻^1^ (SD of change=6.86). Assuming a two-tailed alpha of 0.05 and 80% power, a minimum of 45 participants were required, calculated using a one-sample test on mean change. To reflect the pragmatic, small group-based design, clustering was incorporated using a design effect (DE=1+(*m*−1)×ICC, *m*=10, ICC=0.02–0.05). Aligning with previous exercise oncology research, an additional 20% was incorporated to account for expected attrition [31], resulting in a total sample target of 68–83 participants to detect a clinically meaningful improvement in VO₂peak under real-world delivery conditions.

Descriptive summaries of baseline patient characteristics consisted of frequency distributions for categorical data, and means and standard deviations for continuous measures. Generalized linear mixed effects models (with appropriate canonical links or transformations where appropriate) with participant random effects were utilized to examine pre-post intervention changes in continuous data over time. Results were summarized using estimated marginal means and mean changes with 95% confidence intervals (CI). All models were adjusted for participant diagnosis. Non-parametric tests (Chi-squared test) were used to determine pre-post intervention changes in the Godin Leisure-Time Physical Activity Questionnaire. Associations between VO₂ measures and EORT QLQ-C30 outcomes were examined by including VO₂ variables as covariates in the models for those outcomes. All data were incorporated into the analysis, regardless of missing data points due to loss to follow-up, as maximum likelihood estimation (MLE) methods were utilized in the models. All data were analyzed using Stata version 18.0 (StataCorp, College Station, TX), with significance levels at *p* = 0.05.

## Results

**Fig. 1:**
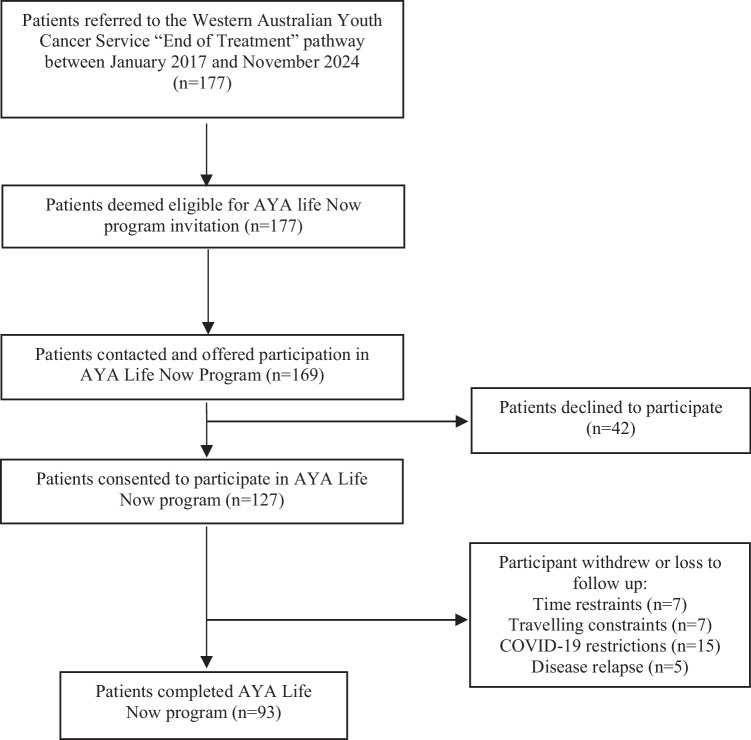
Life Now participant recruitment flowchart for the study

Between January 2017 and November 2024, 127 participants were recruited and completed baseline assessments (Table 1). Over the duration of the 12-week exercise intervention, 34 participants (27%) withdrew or were lost to follow-up for reasons that included time constraints (generally returning to full-time work or study), time and/or financial constraints associated with traveling to the sessions, restrictions due to the COVID-19 pandemic, and disease relapse or progression (Fig. 1).

**Table 1:** Characteristics of participants in the recruited sample

Characteristics	*n* = 127
*Patient demographics*	
Age (years), mean (SD)	21.4 (3.0)
Males, *n* (%)	65 (51.2)
Hematological malignancy,* n* (%)	65 (51.2)
Solid tumor, *n* (%)	61 (48.0)
*Diagnosis*	
Hodgkin lymphoma, *n* (%)	33 (26.0)
Sarcoma, *n* (%)	28 (22.0)
Leukemia, *n* (%)	23 (18.1)
CNS tumor, *n* (%)	9 (7.1)
Other*, *n* (%)	34 (26.8)
*Anthropometrics*	
Height (cm), mean (SD)	173.4 (9.3)
Weight (kg), mean (SD)	72.4 (18.7)
BMI (kg/m^2^), mean (SD)	24.1 (5.9)
Waist girth (cm), mean (SD)	80.7 (14.6)
Hip girth (cm), mean (SD)	99.1 (11.8)

*Other diagnoses include germ cell tumor, breast cancer, colorectal cancer, melanoma, squamous cell carcinoma, thyroid cancer

### Cardiorespiratory fitness and physical function

Of the recruited cohort, 101 participants completed CPET at baseline and post-intervention assessments. The remainder of the sample were deemed ineligible due to medical contraindications, functional limitations, and restrictions due to the COVID-19 pandemic. All CRF and physical functioning data are presented in Table 2. Significant pre-post improvements were observed in predicted VO₂peak (*p* = 0.001) and the 400-m timed walk (*p* < 0.001). Additionally, significant pre-post improvements were also observed in the five-repetition STS test (*p* < 0.001), dominant hand grip strength (*p* = 0.028), maximal push ups (*p* < 0.001) and sit ups (*p* < 0.001), and the 1RM leg press (*p* < 0.001), chest press (*p* < 0.001), and seated row (*p* < 0.001).

**Table 2:** Baseline and 12-week post-intervention outcomes for cardiorespiratory fitness, strength, and functional measures

**Characteristic**	**Baseline**	**Post-intervention**	**Pre-post change**^¥^	***p***
*Cardiorespiratory fitness*				
Predicted VO₂peak (mL kg min^−1^)	30.9 (29.0,32.9)	34.1 (32.0, 36.2)	3.1 (1.00,5.3)	**< 0.001**
400-m timed walk (s)	263.8 (251.5,276.2)	241.6 (229.1, 254.1)	−22.3 (−30.0, −14.6)	** < 0.001**
*Strength*				
1RM leg press (kg)	101.7 (91.7, 111.8)	144.3 (133.7, 154.9)	42.5 (35.0, 50.1)	**< 0.001**
1RM chest press (kg)	32.3 (29.6, 35.1)	40.6 (37.7, 43.5)	8.3 (6.7, 9.8)	** < 0.001**
1RM seated row (kg)	50.6 (47.3, 53.8)	60.4 (57.0, 63.8)	9.8 (7.9, 11.8)	** < 0.001**
*Physical functioning*				
Grip strength (kg)	31.9 (30.3, 33.5)	33 (31.6, 35.1)	1.5 (0.2, 2.8)	**0.028**
Push ups (*n*)	15.7 (14.5, 16.9)	20.0 (18.7, 21.3)	4.3 (3.4, 5.3)	**< 0.001**
Sit ups (*n*)	15.9 (14.8, 17.1)	18.9 (17.6, 20.1)	2.9 (1.7, 4.2)	**< 0.001**
5-rep sit-to-stand (s)	9.6 (9.1, 10.2)	8.5 (7.9, 9.1)	−1.2 (−1.7, −0.6)	**< 0.001**

Shown are means, with 95% confidence intervals

^¥^Adjusted for diagnosis

### Body composition

Body composition data are presented in Table 3. No change in BMC, BMD, or FM was evident over time. However, LMM was significantly higher (*p* < 0.001) and FM% was significantly lower (*p* = 0.002) at 12 weeks compared with baseline.

**Table 3:** Baseline and 12-week post-intervention body composition outcomes

**Characteristic**	**Baseline**	**Post-intervention**	**Pre-post change**^¥^	***p***
Lean muscle mass (total) (g)	44,192 (42,495, 45,890)	45,300 (4359, 47,008)	1108.0 (703.3, 1512.7)	** < 0.001**
Fat mass (total) (g)	24,606 (22,360, 26,853)	24,247 (21,984, 26,510)	−359.6 (−969.4, 250.1)	0.248
Fat mass (percent) (%)	34.3 (32.6, 36.0)	33.6 (31.8, 35.3)	−0.7 (−1.2, −0.3)	**0.002**
Bone mineral content (g)	2400.1 (2306.7, 2493.6)	2428.6 (2334.2, 2523.0)	28.5 (−1.3, 58.3)	0.061
*Z*-score (BMD)	0.45 (0.22, 0.69)	0.49 (0.25, 0.72)	0.03 (−0.04, 0.10)	0.376

Shown are means, with 95% confidence intervals

^¥^Adjusted for diagnosis

### PROMs

Table 4 displays the pre- and post-intervention data for all PROMs. Significant pre-post improvements in EORTC-QLQ-C30 physical functioning (*p* < 0.001), role functioning (*p* < 0.001), social functioning (*p* = 0.001), and fatigue (*p* < 0.001) were observed. Additionally, despite no significant change in emotional (*p* = 0.610) or cognitive functioning (*p *= 0.522) domains, global QoL scores were significantly higher at 12 weeks (*p* < 0.001). Scores were significantly higher at 12 weeks across SF-36 domains: physical functioning (*p* < 0.001), social functioning (*p* < 0.001), fatigue (*p* < 0.001), general health (*p* < 0.001), emotional functioning (*p* < 0.001), and health change (*p* = 0.004). Participants reported a significant increase in their strenuous (*p* < 0.001), moderate (*p* = 0.006), and total (*p* = 0.006) physical activity levels. Significant positive associations between predicted VO₂peak and EORTC-QLQ-C30 physical functioning (*p* = 0.005), fatigue (*p* = 0.035), pain (*p* = 0.046), and global QoL (*p* = 0.041) were evident.

**Table 4:** Baseline and 12-week post-intervention outcomes for EORTC QLQ-C30 and SF-36 domains

**Characteristic**	**Baseline**	**Post-intervention**	**Pre-post change**^¥^	***p***
*EORTC-QLQ-C30*				
Physical	85.7 (83.5, 88.0)	91.5 (89.5, 93.6)	5.8 (4.06, 7.51)	** < 0.001**
Role	76.3 (72.2, 80.4)	86.53 (82.1, 91.0)	10.22 (5.45, 15.00)	** < 0.001**
Emotional	76.8 (72.3, 81.3)	78.2 (73.2, 83.2)	1.4 (−4.0, 6.8)	0.610
Cognitive	68.1 (62.9, 73.3)	69.7 (64.2, 75.3)	1.6 (−3.3, 6.5)	0.522
Social	79.4 (76.0, 83.0)	85.3 (81.8, 88.7)	5.8 (2.2, 9.4)	**0.001**
Fatigue	61.2 (57.6, 64.8)	71.0 (67.1, 74.9)	9.8 (6.03, 13.7)	** < 0.001**
NV	90.9 (88.0, 93.8)	93.3 (90.1, 96.4)	2.38 (−0.6, 5.4)	0.123
Pain	83.4 (80.3, 86.6)	84.8 (81.4, 88.1)	1.4 (−2.1, 4.8)	0.446
QoL	67.0 (63.6, 70.3)	76.8 (73.2, 80.3)	9.8 (6.8, 12.8)	** < 0.001**
*SF-36*				
Physical functioning	73.7 (70.1, 77.3)	84.6 (81.0, 88.7)	11.2 (7.9, 14.4)	** < 0.001**
Role limitations: physical	56.9 (50.2, 63.6)	79.1 (71.6, 86.4)	22.2 (14.6, 29.7)	** < 0.001**
Role limitations: emotional	69.9 (63.6, 76.2)	79.8 (72.9, 86.7)	9.9 (2.7, 17.2)	**0.007**
Fatigue	53.7 (50.3, 57.2)	64.1 (60.5, 67.8)	10.4 (7.0, 13.8)	** < 0.001**
Emotional functioning	71.5 (68.7, 74.3)	77.5 (74.5, 80.5)	6.0 (3.5, 8.5)	** < 0.001**
Social functioning	71.1 (67.3, 74.8)	80.6 (76.5, 84.7)	9.5 (5.3, 13.7)	** < 0.001**
Pain	76.7 (73.2, 80.2)	78.5 (74.7, 82.4)	1.8 (−1.9, 5.6)	0.337
General health	53.2 (50.0, 56.5)	61.1 (57.6, 64.6)	7.9 (4.7, 11.0)	** < 0.001**
Health change	53.41 (46.6, 60.2)	66.6 (59.8, 73.4)	13.2 (4.2, 22.1)	**0.004**

Shown are means, with 95% confidence intervals

^¥^Adjusted fordiagnosis

### Adherence and safety

Adherence to the study protocol was assessed by completion of outcome assessments and attendance at exercise sessions. Overall, 73% of participants completed both pre- and post-intervention assessments, with 34 participants withdrawing before the 12-week follow-up (Fig. 1). Participants attended 86% of scheduled exercise sessions (*m* = 20.7, SD = 3.4). Missed sessions were most often due to viral illness, fatigue, travel, or conflicting appointments. No adverse events were observed during the 12-week intervention period, specifically as a result of the exercise sessions or prescription of exercises.

## Discussion

Exercise is well established as being associated with attenuated treatment-related effects and improved survival outcomes [6, 8, 32]. However, large-scale research involving AYAs remains limited [9, 10], and their distinct physiological and psychosocial needs necessitate caution when generalizing findings from adult or pediatric cohorts. Previous research in AYAs undergoing current treatment has demonstrated promising results in reducing the functional decline associated with treatment, with little insight into the ongoing survivorship challenges faced by this cohort [21]. The current single-arm, pre-post study explored the role of exercise in AYA survivorship, with findings consistent with a beneficial association between the 12-week, group-based program and CRF, functional strength and capacity, and several domains of health-related QoL in this cohort. However, the absence of a control group limits causal inference, and these findings should be considered preliminary, requiring confirmation in randomized controlled trials. To our knowledge, this represents the largest pragmatic study of its kind in this population, addressing key evidence gaps including the paucity of scalable, non-clinical, group-based interventions targeting AYAs with cancer [10].

A significant improvement in CRF, the primary outcome variable, as well as the 400-m timed walk which may be considered a surrogate measure of CRF, was observed following the 12-week exercise intervention. Although the observed 3.1 mL·kg⁻^1^·min⁻^1^ increase in predicted VO₂peak in the current study fell short of the 3.5 mL·kg⁻^1^·min⁻^1^ threshold linked to a clinically significant reduction in all-cause mortality and a reduction in cardiovascular risk, these changes may represent clinically meaningful improvements within a 12-week timeframe [20]. Young people with low CRF face an increased risk of cardiovascular disease, type 2 diabetes, and long-term cancer-related morbidity in survivorship [33, 34]. In support of this primary finding, Atkinson et al. [30] have previously reported significant improvements in this modifiable risk factor (CRF) following an exercise intervention in a relatively small cohort of AYA cancer survivors. Also consistent with prior studies [30, 35], predicted VO₂peak in the current cohort remained below age-matched normative values (39.1 mL·kg⁻^1^·min⁻^1^) [36], which may reflect persistent CRF impairment in AYA survivors. This comparison, however, should be interpreted with caution, as normative values were derived from direct maximal testing, whereas the current study used submaximal extrapolation, which introduces additional estimation error. The secondary outcome measures including patient-reported QoL, fatigue, and functional capacity provided complementary data more directly relevant to patient experience and program evaluation, and demonstrated consistent improvements across these domains. While CRF carries established prognostic significance, future pragmatic trials in this population should consider whether a patient-reported primary outcome would better serve both design intent and the needs of commissioners responsible for program implementation. Collectively, these findings highlight the potential need for early, sustained exercise programs delivered throughout treatment and into long-term survivorship follow-up.

Results from the current study also demonstrated significant pre-post improvements in strength and functional capacity following the exercise program. This is also aligned with previous findings [30, 37]. Strength gains were observed in upper (19% increase in the 1RM seated row; 25% increase in the 1RM chest press) and lower (41% increase in the 1RM leg press) limb strength variables, as well as functional tasks (sit ups, push ups, grip strength, five-repetition STS test). Despite promising improvements, these results similarly reflect a discrepancy with this sample presenting well below age-matched normative data both pre- and post-intervention [38], which may suggest the need for earlier (and also longer) interventions to prevent and/or mitigate this decline. Nevertheless, supporting these strength gains, lean mass was significantly higher and FM% was significantly lower at 12 weeks than at baseline. Given the established association between muscular strength and functional capacity [39, 40], as well as the associations between body composition and comorbid disease, these improvements may be associated with reduced risk of comorbid profiles and improved functional independence in survivorship, though this interpretation requires prospective confirmation [41, 42].

While the deleterious impact of cancer on AYA psychosocial well-being and QoL has been well reported [43–46], limited studies have reported on improvements in these variables associated with exercise in this cohort [9, 47]. Promisingly, the current study observed significantly higher EORTC QLQ-C30 scores in physical, role, and social functioning domains, as well as fatigue and global QoL, following the 12-week program. Each of these domain scores exceeded the minimally important difference threshold of 5 points (range 5.83–10.22 points) [48], suggesting clinically relevant improvements for this cohort. Comparably, SF-36 outcomes also reflected improvements in physical and social functioning, fatigue, and general health, demonstrating consistency across the measures. These findings support previous research in AYA cancer survivors confirming that structured exercise interventions are associated with meaningful improvements in physical and social domains of QoL [10, 21, 30]. The absence of significant changes in emotional or cognitive functioning across both scales in this study should be acknowledged. This lack of change may be reflective of the complex and multifactorial nature of psychosocial recovery in AYAs, which is influenced not only by physical health but also by ongoing treatment effects, pre-morbid or co-morbid psychological considerations, persistent contextual life disruptions, and broader psychosocial stressors [49]. Additionally, this lack of emotional domain change highlights the potential need to consider formal psychological, or behavior change strategies to be included in the research design to elicit benefits for these variables.

The group-based delivery of this program with embedded informal group activities was an intentional design to facilitate unstructured peer interaction. By enabling regular interaction among participants with shared lived experiences, the group-based environment may have been associated with improved social connectedness which is consistently linked to improved psychosocial well-being in AYAs, though this pathway was not directly measured and cannot be confirmed from the current data [50]. This mechanism may also partially explain why the social functioning QoL domains improved in this cohort despite no measurable change in emotional functioning, possibly through a reduction of isolation and fostering the collective shared experience, though these remain speculative interpretations in the absence of process data [51]. Similarly, the group environments may have been associated with motivation, accountability, and adherence, which in turn may relate to the positive functional outcomes observed, but these mechanisms were not formally assessed and should be examined in future work [52]. The positive associations between predicted VO₂peak and multiple QoL domains in this study are consistent with a link between CRF, peer support, and QoL in this cohort. Further exploration of the interaction between group connectedness and psychosocial outcomes in this cohort is warranted.

A stated aim of this study was to support the transition from structured clinical cancer care to independent participation in community-based exercise reflecting real-world survivorship integration. The community gymnasium setting, non-clinical delivery model, and group-based format were selected to reflect the environment and social conditions of routine exercise participation, with the intention of building exercise habits and social connection that may facilitate continued engagement beyond the program. Whether participants sustained exercise engagement following completion of the program was not assessed, and the absence of long-term follow-up data is an acknowledged limitation of the current study. Participants did report significant increases in self-reported physical activity at 12 weeks, which may indicate early behavior change, though whether this reflects durable engagement cannot be determined from these data. Future studies should incorporate follow-up assessments at 6 and 12 months to examine whether program participation is associated with sustained exercise behavior and ongoing survivorship care integration.

### Limitations

While this prospective study was the largest reported cohort of AYA cancer survivors enrolled in a group-based exercise intervention, several limitations must be acknowledged. The single-arm, pre-post design does not include a control group, which is the primary constraint on interpreting these findings: observed pre-post changes cannot be attributed to the program with confidence, and natural recovery, regression to the mean, or other concurrent factors cannot be excluded. The pragmatic design, while reflecting real-world conditions, may also have introduced variability in program delivery, potentially reducing internal validity. The heterogeneous sample with respect to diagnosis and treatment protocols, combined with individualized exercise prescription, may have contributed to outcome variability. Despite improvements across physical and psychological domains, CRF and other physical measures remained below healthy normative reference data. Future studies should examine whether longer program duration is associated with additional benefit. A potential selection bias also warrants consideration, given that participants were recruited from an end-of-treatment pathway and may represent more motivated individuals. This study provides preliminary, real-world evidence to inform the design of future controlled trials in this population.

## Conclusion

This study provides preliminary evidence that participation in a pragmatic, group-based program in a community setting was associated with meaningful improvements in predicted VO₂peak, strength, function, and QoL domains in AYA cancer survivors. The single-arm design limits causal interpretation, and findings require confirmation in randomized controlled trials. Despite ongoing deficits relative to age-matched norms being evident, the observed improvements are consistent with a potentially beneficial role of individualized exercise in addressing persistent treatment-related impairments in this cohort and suggest that early intervention may be warranted. The group-based approach was associated with improvements in social functioning, though the mechanisms underlying this finding remain to be established. Collectively, these findings support the rationale for future controlled trials of early, sustained, and scalable exercise programs extending beyond treatment completion, with particular attention to strategies that may also address emotional and cognitive well-being in AYAs.

## Supplementary Information

Below is the link to the electronic supplementary material.

ESM 1 (DOCX 136 KB)

## Data Availability

Available on request.
