# Supplementary material for: Exercise for survivorship: outcomes associated with a community-based group exercise program for adolescent and young adult cancer survivors
Source: Support Care Cancer. 2026 Jun 6;34(7):622. doi: 10.1007/s00520-026-10838-0 (PMC13242424; doi:10.1007/s00520-026-10838-0)
Supplement: Supplementary file 1 — (DOCX 136 KB) [file 520_2026_10838_MOESM1_ESM.pdf]

Supplementary Material 1 – Safety screening procedures.

Pre-program Medical Clearance

Prior to enrolment, all potential participants will undergo medical clearance via their treating clinician, consistent with pre-exercise screening recommendations for cancer survivors [1, 2]. Clearance will be documented before baseline assessment being scheduled. Participants will be deemed ineligible if any of the following contraindications are present at the time of referral or assessment, in accordance with established absolute contraindications to exercise in cancer rehabilitation [1-3]:

- Medical instability as determined by the treating clinician, including active infection requiring hospitalisation, uncontrolled cardiac arrhythmia, or haemodynamic instability [1].
- Severe anaemia (haemoglobin < 8 g/L) or neutropenia (absolute neutrophil count < 0.5×10^9/L), thrombocytopenia (platelets >50,000 cells/µ) (consistent with haematological safety thresholds recommended for cancer rehabilitation [2].
- Bone metastases at sites conferring fracture risk with moderate-intensity exercise as per the study protocol.
- Absence of medical clearance from the treating clinician for any reason

Participants under 18 years of age will be required to obtain legal guardian consent in addition to their own assent. Medical clearance will be re-confirmed if a participant experienced a significant new health event during the program.

Pre-assessment Safety Screening

Safety screening will be performed by the supervising AEP before each baseline and post-intervention assessment, consistent with recommended pre-exercise evaluation procedures for cancer survivors [1, 3]. The screen will comprise of:

- Verbal review of current symptoms including chest pain, dyspnoea at rest, dizziness, palpitations, and musculoskeletal pain
- Resting heart rate and blood pressure measurement, with assessment deferred if resting systolic blood pressure exceeded 180 mmHg or diastolic blood pressure exceeded 100 mmHg, consistent with cardiovascular precaution thresholds recommended in cancer rehabilitation [4]
- Review of any interim medical events, new diagnoses, or medication changes since the previous assessment
- Confirmation of current haematological status where clinically indicated, based on participant-reported blood results or liaison with the treating team, given the relevance of haematological parameters to exercise safety in this population [2]

Assessment modifications or exclusions will be applied at the AEP's clinical discretion where any criterion is not met. Participants unable to complete CPET due to medical contraindications, functional limitations, or COVID-19 restrictions will complete the 400m timed walk as a surrogate CRF measure.

Pre-session Safety Screening (Exercise Sessions)

Before each of the 24 scheduled exercise sessions, the supervising AEP will conduct a standardised verbal screen with each participant, in accordance with recommended session-by-session monitoring for supervised cancer exercise and rehabilitation programs [1, 2]. This will include:

- Current symptom review: fatigue level (Borg RPE [5]), musculoskeletal pain or discomfort, nausea, dizziness, or new health concerns since the previous session
- Confirmation of absence of fever (> 38.0°C) or active infection symptoms, consistent with recommended infection-related exercise precautions [4]
- Review of any interim medical appointments, investigations, or changes to treatment or medication, including any new corticosteroid use, peripheral neuropathy symptoms, or cardiotoxic treatment exposure relevant to exercise safety.
- Functional readiness assessment, with exercise prescription modified - including reduction in intensity, volume, or mode - or the session deferred and the participant referred to their treating clinician where concerns were identified

Session modifications and reasons will be recorded in the participant's exercise log by the supervising AEP.

Adverse Event Monitoring and Recording

Adverse events are defined as any untoward medical occurrence during or immediately following an assessment or supervised exercise session, including but not limited to: chest pain, syncope or near-syncope, severe dyspnoea, significant musculoskeletal injury, or any event requiring medical attention, consistent with adverse event classifications used in cancer rehabilitation research [1, 2]

The supervising AEP will monitor participants throughout each session and will be trained in emergency first aid and cardiopulmonary resuscitation. The gymnasium has AED available on-site. The research team includes a senior cancer clinician who provides guidance on individual safety concerns throughout the study, reflecting the multidisciplinary oversight recommended for exercise programs in cancer populations [1]. Regular research team meetings will be held to review protocol adherence and safety reports. Adverse events will be recorded with documentation of the nature and severity of the event, time of onset, action taken, and clinical outcome. SAEs - defined as any event resulting in hospitalisation,

prolonged disability, or considered life-threatening, will be reported to the Sir Charles Gairdner Hospital HREC (RGS0000001819) within an expedient timeframe. In the event of frequent adverse events deemed attributable to the intervention, a collaborative decision between the research team and senior clinician will be made to cease the trial. Any protocol modifications will be submitted to and approved by the relevant HREC prior to implementation. All data will be collected, entered, and stored in accordance with HREC guidelines.

References

1. Hayes, S.C., et al., The Exercise and Sports Science Australia position statement: Exercise medicine in cancer management. Journal of science and medicine in sport, 2019.
2. Maltser, S., et al., A focused review of safety considerations in cancer rehabilitation. PM&R, 2017. 9(9): p. S415-S428.
3. Campbell, K.L., et al., Exercise guidelines for cancer survivors: consensus statement from international multidisciplinary roundtable. Medicine & Science in Sports & Exercise, 2019. 51(11): p. 2375-2390.
4. Santa Mina, D., et al., Exercise as part of routine cancer care. The Lancet Oncology, 2018. 19(9): p. e433-e436.
5. Borg, G., Borg's perceived exertion and pain scales. 1998: Human kinetics.
